# Association Between Self-Perceived Stress and Cryptogenic Ischemic Stroke in Young Adults: A Case-Control Study

**DOI:** 10.1212/WNL.0000000000213369

**Published:** 2025-03-04

**Authors:** Shakar Kutal, Lauri Juhani Tulkki, Tomi Sarkanen, Petra Redfors, Katarina Jood, Annika Nordanstig, Nilüfer Yeşilot, Mine Sezgin, Pauli Ylikotila, Marialuisa Zedde, Ulla Junttola, Annette Fromm, Kristina Ryliskiene, Radim Licenik, Phillip Ferdinand, Dalius Jatužis, Liisa Kõrv, Janika Kõrv, Alessandro Pezzini, Juha Sinisalo, Mika Lehto, Eva Gerdts, Jaana Autere, Ana Catarina Fonseca, Ulrike Waje-Andreassen, Bettina Von Sarnowski, Tiina Sairanen, Turgut Tatlisumak, Juha Huhtakangas, Pekka Jäkälä, Jukka Putaala, Nicolas Martinez-Majander

**Affiliations:** 1 Neurology, Helsinki University Hospital, and University of Helsinki, Finland;; 2 Department of Neurology, Tampere University Hospital, Wellbeing Services County of Pirkanmaa, Finland and Faculty of Medicine and Health Technology, Tampere University;; 3 The Sahlgrenska Academy at University of Gothenburg and Department of Neurology, Institute of Neuroscience and Physiology, Sahlgrenska University Hospital, Sweden;; 4 Istanbul Faculty of Medicine, Department of Neurology, Istanbul University, Turkey;; 5 Department of Neurology, Neurocenter, Turku University Hospital, University of Turku, Finland;; 6 Neurology Unit, Azienda Unità Sanitaria Locale–IRCCS di Reggio Emilia, Italy;; 7 Department of Neurology, Faculty of Medicine, Neurocenter, Oulu University Hospital, Finland and Research Unit of Clinical Medicine, University of Oulu;; 8 Department of Neurology, Haukeland University Hospital, Bergen, Norway;; 9 Centre of Neurology, Faculty of Medicine, Vilnius University, Lithuania;; 10 North West Anglia NHS Foundation Trust Acute Stroke Centre, United Kingdom;; 11 Neurosciences, University Hospitals of North Midlands NHS Trust, Stoke-on-Trent, United Kingdom;; 12 Department of Neurology and Neurosurgery, University of Tartu, Estonia;; 13 Department of Medicine and Surgery, Department of Emergency, Parma University Hospital, University of Parma and Stroke Care Program, Italy;; 14 Department of Cardiology, Helsinki University Hospital and University of Helsinki, Finland;; 15 Department of Internal Medicine, Jorvi Hospital, HUS Helsinki University Hospital, and University of Helsinki, Finland;; 16 Department of Heart Disease, Haukeland University Hospital, Bergen, Norway;; 17 Department of Clinical Science, University of Bergen, Norway;; 18 Neurocenter Neurology, Kuopio University Hospital, Finland and University of Eastern Finland;; 19 Department of Neurosciences and Mental Health (Neurology), Hospital de Santa Maria-CHLN, Faculdade de Medicina, Universidade de Lisboa, Portugal; and; 20 Department of Neurology, University Medicine Greifswald, Germany.

## Abstract

**Background and Objectives:**

Psychosocial stress is a potentially modifiable risk factor of early-onset ischemic stroke, with limited evidence suggesting a stronger association between stress and cryptogenic ischemic stroke (CIS) compared with strokes of known etiology. We aimed to explore the association between self-perceived stress and CIS, with subgroup analyses stratified by sex and age.

**Methods:**

Young patients aged 18–49 years with a first-ever CIS and sex-matched and age-matched stroke-free controls from 19 European centers were included. Self-perceived stress was assessed using a modified version of the Perceived Stress Scale (PSS). Scores were categorized into low (0–13), moderate (14–26), and high (27–40) perceived stress. Conditional logistic regression—adjusted for age, level of education, traditional risk factors (hypertension, cardiovascular diseases, diabetes mellitus, heavy alcohol consumption, current smoking, obesity, diet, depression, and physical inactivity), and migraine with aura (MA)—was used to assess independent association between self-perceived stress and CIS.

**Results:**

Altogether, 426 patients (median age 41 years; 47.7% women) and 426 controls were included. Patients were more often at least moderately stressed compared with controls (46.2% vs 33.3%, *p* < 0.001). In the entire study population, higher self-perceived stress as a discrete measure was independently associated with CIS: adjusted odds ratio (OR) 1.04 per point increase; 95% CI 1.01–1.07. Categorical PSS score analysis showed an independent association between moderate stress and CIS (OR 1.47; 95% CI 1.00–2.14), but not with high stress (2.62; 0.81–8.45). In sex-specific analysis, higher stress as a discrete measure was associated with CIS in women (1.06; 1.02–1.11), but not in men (1.02; 0.97–1.07). Moderate stress was associated with CIS in women (1.78; 1.07–2.96), but not in men (1.06; 0.58–1.96). When stratified by age, higher stress as a discrete measure was significantly associated with CIS only in patients aged 18–39 years (1.06; 1.00–1.11).

**Discussion:**

Self-perceived stress was strongly correlated with an increased risk of early-onset CIS, even after robust adjustment for cardiovascular risk factors and MA. These findings highlight the need for further investigation into the mechanisms by which stress may contribute to the risk of CIS. Possibility of recall bias should be considered when interpreting the results.

**Trial Registration Information:**

Clinical trial registration number: NCT01934725.

## Introduction

Over the past decade, extensive research has shown a significant prevalence of traditional vascular risk factors in young patients experiencing ischemic stroke (IS), alongside a noticeable rise in the incidence of early-onset IS.^1-3^ However, there is compelling evidence suggesting that habitual risk factors might play a more substantial role in the development of IS among younger individuals compared with older ones.^4^ Indeed, a German nationwide case-control study identified 4 traditional risk factors that had the strongest contribution to early-onset IS, and three of them were habitual.^5^ Low physical activity and hypertension were most strongly associated with early-onset IS, followed by smoking and heavy episodic alcohol consumption.^5^ Other studies have shown an association between migraine with aura (MA), heavy alcohol consumption, and a subgroup of patients with early-onset cryptogenic ischemic stroke (CIS).^6,7^ Notably, a recent prospective population-based study highlighted that an increasing number of early-onset IS cases occur in individuals without traditional vascular risk factors and with undetermined causes, suggesting that other factors may be contributing to this increasing trend.^8^

Stress could be one of the factors explaining IS in modern times, especially among younger individuals, due to the increasing demands and pressures associated with work, including long hours, job insecurity, and high expectations. In addition, family matters and financial burdens can also contribute significantly to chronic stress. Consequently, the relationship between self-perceived stress and IS at young age has gained increasing scientific interest.^9-12^ In a Swedish study, persistent self-perceived psychological stress was associated with a 3.5-fold increased risk of IS in patients aged 18–69 years, CIS showing the highest point estimate among stroke subtypes for this association with an odds ratio (OR) of 4.03.^9^ In another study, analysis of young West Africans with IS reported a 3-fold increased risk of self-perceived stress in the preceding 2 weeks before the IS.^10^

There is growing interest in exploring less well-documented risk factors of early-onset CIS because traditional vascular risk factors often fail to fully explain this subtype. These emerging factors may also play a key role in the rising incidence of IS in younger populations. Therefore, we aimed to explore the association between self-perceived stress and early-onset CIS, with subgroup analyses stratified by sex and age.

## Methods

### Study Population

From November 2013 to November 2022, a total of 546 young patients with CIS and 546 stroke-free control participants matched by age and sex were recruited across 19 European centers as part of the prospective multicenter Searching for Explanations for Cryptogenic Stroke in the Young: Revealing the Etiology, Triggers, and Outcome (SECRETO, NCT01934725) study. The study included patients aged 18–49 years who were hospitalized for a first-ever imaging-confirmed CIS, and all participants were assessed using a standardized protocol previously outlined.^13^ Patients were included if an acute ischemic lesion by diffusion-weighted imaging in MRI or an arterial occlusion and perfusion deficit correlating with acute symptoms were demonstrated. Silent brain infarcts or previous transient ischemic attacks were allowed. Patients were excluded if baseline minimum tests (including brain MRI and routine blood tests) were not obtained within the first week after stroke onset or if additional diagnostic tests (such as imaging of cervicocephalic arteries, echocardiography (ECG), 24-hour Holter monitoring, or thrombophilia screening) were not performed within the first 2 weeks.

All enrolled patients underwent a comprehensive and standardized diagnostic workup to rule out definitive causes of stroke. The evaluation included brain MRI, imaging of both extracranial and intracranial vessels using either CT angiography or magnetic resonance angiography, and laboratory tests according to the study protocol. In addition, a 12-lead ECG and continuous ECG monitoring for a minimum of 24 hours were performed, alongside both transthoracic echocardiogram and transesophageal echocardiogram (TEE), which were conducted following a standardized protocol.^14^ Individual coagulopathies were not considered as exclusion criteria because of uncertain causality, with the exception of a previously diagnosed antiphospholipid antibody syndrome. Additional diagnostic tests, such as transcranial Doppler (TCD) ultrasound with bubble test, were performed at selected study sites. Stroke severity was assessed using the NIH Stroke Scale (NIHSS) score.

CIS was defined based on the A-S-C-O classification, specifically as the absence of disease (grade 0) or as having a grade II (causality uncertain) or grade III (unlikely to be a direct cause) pathology, determined using diagnostic tests with the highest available level of evidence.^15^

For each patient, 1 stroke-free control matched by sex and age (±5 years) from the same region was identified locally at each study center. Controls were sourced through various methods, including random selection from population registers when feasible, recruitment of patients' unrelated proxies, and hospital staff members who were not involved in the study. Owing to variations in legislation across study sites, the methods for identifying control participants were not uniform. Controls were eligible if they had no history of stroke, assessed with the Questionnaire for Verifying Stroke-Free Status.^16^ Controls were preferably recruited within 3 months of patient enrollment to minimize the potential impact of temporal confounders.

### Standard Protocol Approvals, Registrations, and Patient Consents

The study protocol was approved by the local ethics committees at each recruiting site. All participants gave a written informed consent. Data are available on reasonable request. This study adheres to Strengthening the Reporting of Observational Studies in Epidemiology reporting guidelines.

### Cardiovascular Risk Factors and Comorbidities

A comprehensive clinical history was gathered from all participants through medical records and a structured interview conducted during the study visit. Low education level was classified as primary or lower secondary education, or upper secondary education. Cardiovascular risk factors documented included hypertension (evidenced by a previous diagnosis, previous antihypertensive treatment, or an average blood pressure reading ≥140/90 from 2 office measurements), diabetes mellitus (indicated by a previous diagnosis or antidiabetic medication), hypercholesterolemia (evidenced by a previous diagnosis or use of lipid-lowering medications), and cardiovascular disease (such as coronary artery disease, congestive heart failure, or peripheral arterial disease). None of the participants presented with atrial fibrillation. In addition, current tobacco use (smoking in average of at least one cigarette per day), waist-to-hip ratio (obesity defined as >0.85 in women and >0.90 in men), an unhealthy diet, physical inactivity, and heavy alcohol consumption were recorded. Physical inactivity was determined using the short form of the International Physical Activity Questionnaire,^17^ defined as failing to meet the criteria for at least 1,500 metabolic equivalents per week. Participants' dietary habits were assessed using a modified version of the Mediterranean Diet Score, where higher scores represented healthier diet, and a median cutoff of 24 points was used to create a dichotomous variable.^18^ The World Health Organization Alcohol, Smoking, and Substance Involvement Screening Test was adapted and used to evaluate alcohol consumption through a structured interview conducted with both patients and controls.^7,19^ Depression was defined as experiencing feelings of sadness, low mood, or depression lasting for 2 or more consecutive weeks within the past year.^20^ MA was determined using a validated migraine screener.^6^ The migraine screening tool consists of 9 questions, including 3 initial screening questions regarding headache attacks and attacks with visual aura, followed by 6 detailed questions addressing specific headache characteristics.

In this study, we used a modified version of the Perceived Stress Scale (PSS)^21^ to provide a standardized measure of self-perceived stress levels among both patients with CIS and stroke-free controls during the past month. PSS is a widely recognized questionnaire that consists of 10 questions designed to evaluate the degree to which individuals perceive their lives as stressful. For patients with CIS, the questionnaire was administered after their stroke, but they were instructed to assess their perceived stress levels based on their experiences in the past month before IS. The responses were scored, with total scores categorized into 3 distinct levels: 0–13 points indicating low perceived stress, 14–26 points indicating moderate perceived stress, and 27–40 points indicating high perceived stress.^22-24^ In addition, to provide a more specific assessment of the types and frequency of stress experienced by participants, we used a brief three-item questionnaire to assess stress related to work, home, and financial situations within 12 months preceding the stroke. Stress was defined as experiencing irritability, anxiety, or sleep difficulties due to circumstances at work, those at home, or financial concerns.^25^ For the questions on work and home stress, participants were asked to indicate the frequency of their stress using the following response options: 1: never; 2: some periods; 3: several periods; or 4: permanent stress. Financial stress levels were categorized as follows: 1: little or none; 2: moderate; or 3: high or severe.

For this study, a clinically relevant high-risk patent foramen ovale (PFO) in patients was identified by the presence of PFO in conjunction with an atrial septal aneurysm observed by TEE, or by the detection of a large shunt through either TEE or TCD bubble study.^14,26^

### Statistical Analysis

For variables with more than 10 missing values, including waist circumference (8.4% missing), hip circumference (8.7% missing), and diet score (14.9% missing), data imputation was conducted using multivariable imputation with chained equations through the R package. No imputation was necessary for control variables because none had more than 10 missing values. Frequency of missing values was reported.

Univariable comparisons of baseline characteristics between patients and controls were conducted using statistical tests suited for matched case-control studies. The McNemar test was applied for dichotomous variables, the paired *t* test was used for normally distributed continuous or discrete variables, and the Wilcoxon signed-rank test was used for non-normally distributed continuous variables. Results are reported as absolute numbers with percentages, means with standard deviations (SDs), or medians with interquartile ranges (IQRs). A *p* value of less than 0.05 was deemed statistically significant.

To address any potential imbalances between patients and controls, conditional logistic regression analysis appropriate for matched case-control studies was used. The adjusted ORs and 95% CIs were reported across 3 models: (1) analysis adjusted for age and level of education; (2) analysis adjusted for age, level of education, and predefined vascular risk factors; and (3) fully adjusted analysis including age, level of education, predefined vascular risk factors, and MA. Predefined risk factors, selected based on previous literature, included hypertension, diabetes mellitus, current smoking, obesity, physical inactivity, unhealthy diet, heavy alcohol use, depression, and other cardiovascular diseases.

Additional conditional logistic regression analyses with similar models were performed to explore the association stratified by sex for PSS score, stress at work, stress at home, or financial stress with CIS. As an exploratory analysis, these analyses were also performed for 2 predetermined age groups (18–39 and 40–49 years).

To evaluate the robustness of the results, we conducted a sensitivity analysis by comparing patients with controls sourced exclusively from population-based registries as described above, thereby excluding controls recruited from hospital staff and unrelated proxies.

Statistical analyses were performed with International Business Machines Statistical Package for the Social Sciences Statistics for Windows, version 29.0 (IBM Corp., Armonk, NY), and RStudio, version 2024.04.2 + 764 (RStudio Team, 2024).

## Results

Of the initial 546 matched case-control pairs, we included 426 patients with CIS (median age 41 years, IQR 34–46; 47.7% women) and 426 age-matched and sex-matched controls who had completed the PSS questionnaire. Among patients, the median time from symptom onset to hospital admission was 0 days (IQR 0–1) and the median delay from hospital admission to study inclusion/interview was 6 days (IQR 5–9). The median NIHSS score on admission was 2 (IQR 0–4, range 0–35). Of all patients, 25.9% had NIHSS score 0, 52.7% had mild strokes (NIHSS scores 1–4), 12.2% had moderate strokes (NIHSS scores 5–9), and 9.2% had severe strokes (NIHSS score ≥10). Stroke severity did not differ between patients with low, moderate, and high levels of stress (mild strokes 55.7% vs 48.6% vs 55.6%, moderate strokes 11.0% vs 13.4% vs 16.7%, and severe strokes 7.5% vs 11.7% vs 5.6%, respectively, *p* = 0.657).

### Univariable Comparison Between Patients and Matched Controls

**Figure 1:**
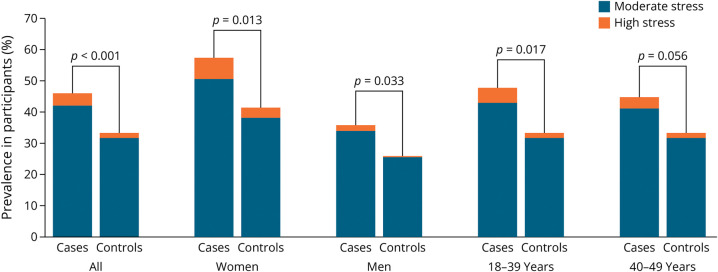
Comparison of Different Perceived Stress Scale Categories for All Study Participants and Stratified by Sex and Age Group

**Figure 2:**
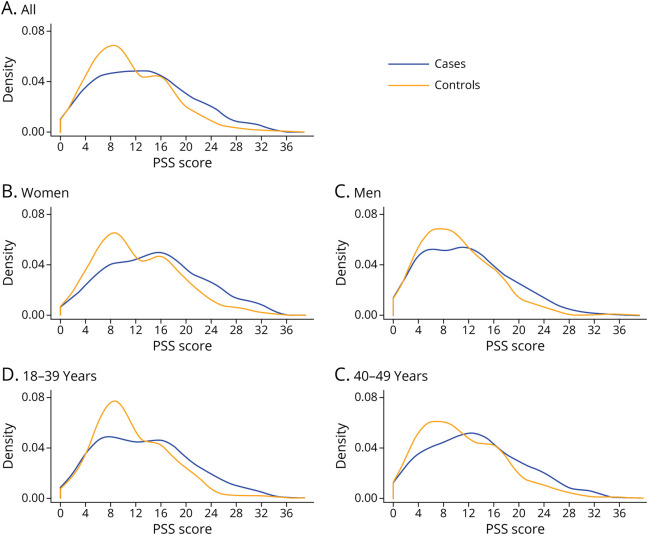
Density of Different Perceived Stress Scale Scores in Cases and Controls, Also Stratified by Sex and Age Group

Apart from stress measures, clinical characteristics of patients with CIS and stroke-free controls are presented in Table 1. Compared with controls, patients exhibited a higher prevalence of low level of education, hypertension, current smoking, abdominal obesity, physical inactivity, depression, unhealthy diet, and MA. Regarding stress measures, in the entire study population, patients were more likely to perceive themselves stressed compared with controls (Table 2, Figures 1 and 2).

**Table 1:** Baseline Characteristics of Young Cryptogenic Ischemic Stroke Cases and Stroke-Free Controls Included in the Study

Characteristic (no. of cases/controls with missing data)	All			Women			Men		
	Cases (n = 426)	Controls (n = 426)	*p* Value	Cases (n = 203)	Controls (n = 203)	*p* Value	Cases (n = 223)	Controls (n = 223)	*p* Value
Age	41 (34–46)	41 (33–46)	N/A	40 (30–45)	40 (30–45)	N/A	42 (36–46)	42 (35–47)	N/A
Low level of education (1/0)	228 (53.6)	150 (35.2)	<0.001	103 (51.0)	67 (33.0)	<0.001	125 (56.1)	83 (37.2)	<0.001
Hypertension (0/3)	145 (34.0)	113 (26.7)	0.019	63 (31.0)	41 (20.4)	0.017	82 (36.8)	72 (32.4)	0.358
Diabetes mellitus (0/1)	15 (3.5)	7 (1.6)	0.134	5 (2.5)	0 (0.0)	N/A	10 (4.5)	7 (3.2)	0.629
Cardiovascular disease^a^	4 (0.9)	3 (0.7)	1.000	1 (0.5)	0 (0.0)	N/A	3 (1.3)	3 (1.3)	1.000
Current tobacco smoking (2/1)	135 (31.8)	60 (14.1)	<0.001	56 (27.7)	28 (13.8)	<0.001	79 (35.6)	32 (14.4)	<0.001
Hypercholesterolemia	6 (1.4)	20 (4.7)	0.004	1 (0.5)	4 (2.0)	0.375	5 (2.2)	16 (7.2)	0.013
Physical inactivity (1/3)	120 (28.2)	100 (23.6)	0.142	63 (31.0)	48 (23.9)	0.148	57 (25.7)	52 (23.4)	0.620
Obesity^b^ (0/5)	244 (57.3)	191 (45.4)	<0.001	81 (39.9)	51 (25.4)	0.002	163 (73.1)	140 (63.6)	0.022
Heavy alcohol use (3/1)	50 (11.8)	25 (5.9)	0.002	22 (10.9)	13 (6.4)	0.108	28 (12.6)	12 (5.4)	0.009
Depression (0/3)	134 (31.5)	102 (24.1)	0.022	74 (36.5)	60 (29.7)	0.184	60 (26.9)	42 (19.0)	0.064
Unhealthy diet (0/1)	217 (50.9)	158 (37.2)	<0.001	96 (47.3)	60 (29.7)	<0.001	121 (54.3)	98 (43.9)	0.042
Obstructive sleep apnea	8 (1.9)	12 (2.8)	0.503	1 (0.5)	3 (1.5)	0.625	7 (3.1)	9 (4.0)	0.804
Migraine with aura	180 (42.3)	67 (15.7)	<0.001	103 (50.7)	44 (21.7)	<0.001	77 (34,5)	23 (10.3)	<0.001

Abbreviation: N/A = not applicable.

Data are n (%) or median (interquartile range).

a Cardiovascular disease includes any of the following: coronary heart disease, chronic heart failure, peripheral artery disease, or history of myocardial infarction.

b Waist-to-hip ratio: >0.85 in women, >0.90 in men.

**Table 2:** Comparison of Stress Assessment Between Young Cryptogenic Ischemic Stroke Cases and Stroke-Free Controls in the Entire Cohort and by Sex

Characteristics (no. of cases/controls with missing data)	All			Women			Men		
	Cases (n = 426)	Controls (n = 426)	*p* Value	Cases (n = 203)	Controls (n = 203)	*p* Value	Cases (n = 223)	Controls (n = 223)	*p* Value
Perceived Stress Scale score^a^	13 (7–18)	10 (7–15)	<0.001	15 (9–20)	11 (7–17)	<0.001	11 (6–15)	9 (6–14)	0.018
At least moderate stress	197 (46.2)	142 (33.3)	<0.001	117 (57.6)	84 (41.4)	0.002	80 (35.9)	58 (26.0)	0.026
High perceived stress	18 (4.2)	7 (1.6)	0.043	14 (6.9)	6 (3.0)	0.115	4 (1.8)	1 (0.4)	0.375
Stress at work (2/0)									
Several periods or permanent	172 (40.6)	163 (38.3)	0.565	89 (43.8)	86 (42.4)	0.838	83 (37.6)	77 (34.5)	0.614
Permanent	60 (14.2)	41 (9.6)	0.061	34 (16.7)	20 (9.9)	0.054	26 (11.8)	21 (9.4)	0.560
Stress at home (7/0)									
Several periods or permanent	103 (24.6)	64 (15.0)	<0.001	60 (30.2)	40 (19.7)	0.018	43 (19.5)	24 (10.8)	0.011
Permanent	15 (3.6)	10 (2.3)	0.405	10 (5.0)	6 (3.0)	0.454	5 (2.3)	4 (1.8)	1.000
Financial stress (4/0)									
Moderate or severe	195 (46.2)	153 (35.9)	0.002	95 (47.3)	70 (34.5)	0.014	100 (45.2)	83 (37.2)	0.070
Severe	44 (10.4)	29 (6.8)	0.077	24 (11.9)	20 (9.9)	0.618	20 (9.0)	9 (4.0)	0.052
Any type of stress (7/0)^b^	220 (52.5)	198 (46.5)	0.082	113 (56.8)	112 (55.2)	0.749	107 (48.6)	86 (38.6)	0.048
Sum of stress categories >3 (7/0)^c^	156 (37.2)	113 (26.5)	0.001	80 (40.2)	60 (29.6)	0.038	76 (34.5)	53 (23.8)	0.021

Data are n (%) or median (interquartile range).

a Low stress: 0–13; moderate stress: 14–26; high perceived stress: 27–40.

b Includes cases and controls that had either several periods of stress or permanent stress at work or home, or severe financial stress.

c Scoring for stress at work or home: 0 = never, 1 = some of the time, 2 = several periods, and 3 = permanent. Scoring for financial stress: 0 = little or none, 1 = moderate, and 2 = severe.

Female patients had a lower level of education and unhealthier diet compared with female controls. They were also more likely to be current smokers, obese, and hypertensive and to have MA. Female patients scored higher on the PSS (15, IQR 9–20) compared with female controls (11, IQR 7–17, *p* < 0.001). There was a significant difference in at least moderate self-perceived stress, several periods of stress or permanent stress at home, and moderate or severe financial stress. Using the sum of different stress categories variable (Table 2), there was a significant difference in female patients compared with controls.

Male patients showed a lower level of education and had unhealthier diet compared with male controls. They were more often obese, heavy alcohol users, and current smokers and had more MA (Table 1). Male patients also scored higher on the PSS (11, IQR 6–15) compared with controls (9, IQR 6–14, *p* = 0.018). Similar to female patients, male patients had a significant difference in at least moderate self-perceived stress and in several periods of stress or permanent stress at home. When evaluating any type of stress for men (Table 2), patients exhibited significantly more stress compared with male controls. In addition, evaluating the sum of different stress categories provides a significant difference in male patients compared with male controls.

In the age-specific comparisons, patients aged younger than 40 years scored higher on the PSS than their age-matched controls. In this subgroup, there was also a significant difference in at least moderate self-perceived stress, several periods of stress or permanent stress at home, moderate or severe financial stress, and sum of stress categories when patients were compared with controls (Table 3). Among those aged 40–49 years, a significant difference was discovered in those having at least moderate stress, and PSS score as a discrete measure (Table 3).

**Table 3:** Comparison of Stress Assessment Between Young Cryptogenic Ischemic Stroke Cases and Stroke-Free Controls in the Entire Cohort and by Sex, Stratified by Age

Characteristics (no. of cases/controls with missing data)	Age younger than 40 y			Age 40 y and older		
	Cases (n = 188)	Controls (n = 188)	*p* Value	Cases (n = 238)	Controls (n = 238)	*p* Value
Perceived Stress Scale score^a^	13 (7–18)	10 (7–15)	0.003	12 (7–18)	10 (6–15)	<0.001
At least moderate stress	90 (47.9)	63 (33.5)	0.004	107 (45.0)	79 (33.2)	0.013
High perceived stress	9 (4.8)	3 (1.6)	0.146	9 (3.8)	4 (1.7)	0.267
Stress at work (2/0)						
Several periods or permanent	75 (39.9)	81 (43.1)	0.586	97 (41.1)	82 (34.5)	0.189
Permanent	28 (14.9)	20 (10.6)	0.312	32 (13.6)	21 (8.8)	0.135
Stress at home (7/0)						
Several periods or permanent	53 (28.6)	30 (16.0)	0.003	50 (21.4)	34 (14.3)	0.060
Permanent	8 (4.3)	5 (2.7)	0.549	7 (3.0)	5 (2.1)	0.774
Financial stress (4/0)						
Moderate or severe	98 (52.4)	68 (36.2)	0.002	97 (41.3)	85 (35.7)	0.202
Severe	24 (12.8)	14 (7.4)	0.110	20 (8.5)	15 (6.3)	0.473
Any type of stress (7/0)^b^	98 (53.0)	96 (51.1)	0.668	122 (52.1)	102 (42.9)	0.062
Sum of stress categories >3 (7/0)^c^	76 (41.1)	53 (28.2)	0.010	80 (34.2)	60 (25.2)	0.062

Data are n (%) or median (interquartile range).

a Low stress: 0–13; moderate stress: 14–26; high perceived stress: 27–40.

b Includes cases and controls that had either several periods of stress or permanent stress at work or home, or severe financial stress.

c Scoring for stress at work or home: 0 = never, 1 = some of the time, 2 = several periods, and 3 = permanent. Scoring for financial stress: 0 = little or none, 1 = moderate, and 2 = severe.

### Association Between PSS Score and CIS

After adjusting for age and level of education, a significant association between PSS score and early-onset CIS in the entire study population emerged in all PSS categories, which included moderate stress, high stress, and PSS score as a discrete measure. These associations remained significant after adjusting for further vascular risk factors and MA, except for high stress (Table 4).

**Table 4:** Odds Ratios and 95% CIs From Conditional Logistic Regression Models on the Association Between Stress Measured With the Perceived Stress Scale (PSS) Score and Cryptogenic Ischemic Stroke, Also Stratified by Sex

	Model 1: Adjusted for age and level of education		Model 2: Adjusted for age, level of education, and predefined vascular risk factors^a^		Model 3: Adjusted for age, level of education, predefined vascular risk factors, and migraine with aura^a,b^	
	OR (95% CI)	*p* Value	OR (95% CI)	*p* Value	OR (95% CI)	*p* Value
All (426 pairs)						
PSS score categories^c^						
Low stress	Reference		Reference		Reference	
Moderate stress	1.71 (1.26–2.31)	<0.001	1.48 (1.05–2.08)	0.027	1.47 (1.00–2.14)	0.048
High stress	2.90 (1.15–7.32)	0.024	2.54 (0.89–7.23)	0.080	2.62 (0.81–8.45)	0.107
PSS, per 1-point increment	1.05 (1.03–1.08)	<0.001	1.05 (1.02–1.08)	<0.001	1.04 (1.01–1.07)	0.007
Women (203 pairs)						
PSS score categories^c^						
Low stress	Reference		Reference		Reference	
Moderate stress	1.73 (1.15–2.62)	0.009	1.70 (1.07–2.70)	0.024	1.78 (1.07–2.96)	0.026
High stress	2.45 (0.88–6.82)	0.087	2.69 (0.79–9.13)	0.112	2.56 (0.70–9.38)	0.155
PSS, per 1-point increment	1.05 (1.02–1.08)	0.001	1.06 (1.02–1.10)	0.002	1.06 (1.02–1.11)	0.005
Men (223 pairs)						
PSS score categories^c^						
Low stress	Reference		Reference		Reference	
Moderate stress	1.67 (1.05–2.62)	0.030	1.17 (0.69–2.00)	0.565	1.06 (0.58–1.96)	0.850
High stress	5.83 (0.56–60.16)	0.139	4.01 (0.35–45.95)	0.265	5.05 (0.16–159.51)	0.358
PSS, per 1-point increment	1.06 (1.02–1.09)	0.004	1.03 (1.00–1.08)	0.145	1.02 (0.97–1.07)	0.499

Abbreviation: OR = odds ratio.

a Predefined vascular risk factors include hypertension, diabetes, current smoking, obesity, physical inactivity, unhealthy diet, heavy alcohol use, and depression. Diabetes was left out from women because of very low prevalence.

b P for interaction between PSS score and sex: 0.480.

c Low stress: 0–13; moderate stress: 14–26; high perceived stress: 27–40.

### Association Between PSS Score and CIS According to Sex

In sex-specific analysis, the association between PSS score and CIS was significant in women after adjusting for age and level of education. The association remained significant in moderate stress and PSS score as a discrete measure, but not in high stress when adjusted for further vascular risk factors and MA (Table 4).

Among men, moderate stress and PSS score per 1-point increment had a significant association with CIS when adjusted for age and level of education. After adjusting for further vascular risk factors and MA, we observed no significant association with any of the stress variables (Table 4). While the point estimate was higher among women, there was no statistically significant interaction between PSS score and sex (*p* = 0.480).

### Association Between PSS Score and CIS According to Age Group

Among those aged 18–39 years, significant association was observed in the model adjusting for age and level of education. After adjusting for vascular risk factors and MA, the association remained significant only for PSS score as a discrete measure (Table 5). For those aged 40–49 years, significant association was observed after adjusting for demographics, but not in the fully adjusted model (Table 5).

**Table 5:** Odds Ratios and 95% CIs From Conditional Logistic Regression Models on the Association Between Stress Measured With the Perceived Stress Scale (PSS) Score and Cryptogenic Ischemic Stroke, Stratified by Age

	Model 1: Adjusted for age and level of education		Model 2: Adjusted for age, level of education, and predefined vascular risk factors^a^		Model 3: Adjusted for age, level of education, predefined vascular risk factors, and migraine with aura^a,b^	
	OR (95% CI)	*p* Value	OR (95% CI)	*p* Value	OR (95% CI)	*p* Value
Age 18–39 y (188 pairs)						
PSS score categories^c^						
Low stress	Reference		Reference		Reference	
Moderate stress	2.06 (1.21–3.50)	0.007	1.71 (0.92–3.18)	0.092	1.86 (0.95–3.64)	0.069
High stress	4.82 (1.12–20.71)	0.035	4.34 (0.75–25.14)	0.102	3.04 (0.49–18.76)	0.232
PSS, per 1-point increment	1.06 (1.02–1.11)	0.002	1.06 (1.01–1.11)	0.026	1.06 (1.00–1.11)	0.042
Age 40–49 y (238 pairs)						
PSS score categories^c^						
Low stress	Reference		Reference		Reference	
Moderate stress	1.71 (1.26–2.31)	<0.001	1.48 (1.05–2.08)	0.027	1.27 (0.78–2.06)	0.334
High stress	2.90 (1.15–7.32)	0.024	2.54 (0.89–7.23)	0.080	1.94 (0.38–9.96)	0.427
PSS, per 1-point increment	1.05 (1.02–1.08)	0.002	1.04 (1.01–1.08)	0.022	1.03 (0.99–1.07)	0.118

Abbreviation: OR = odds ratio.

a Predefined vascular risk factors include hypertension, diabetes, current smoking, obesity, physical inactivity, unhealthy diet, heavy alcohol use, and depression.

b P for interaction between PSS score and age group: 0.660.

c Low stress: 0–13; moderate stress: 14–26; high perceived stress: 27–40.

### Association Between Stress Categories and CIS

For the entire cohort, when evaluating alternative stress categories, in the model adjusting for demographics, several significant associations rise. When adjusted for all vascular risk factors, significant association was found in several periods of stress at home and sum of stress categories (eTable 1).

Sex-specific analysis revealed that among women, the only statistically significant association in the fully adjusted model was observed for permanent stress at work (eTable 2). By contrast, among men, independent associations were not observed in any of the categories (eTable 3).

For those aged 18–39 years, the fully adjusted model showed significant associations for stress over multiple periods or permanent home-related stress, severe financial stress, and the sum of stress categories (eTable 4). In those aged 40–49 years, significant associations in the fully adjusted model were not observed in any categories (eTable 5).

### Sensitivity Analyses

When selecting case-control pairs with strictly population-based controls (n = 250), higher self-perceived stress was still associated with early-onset CIS in the entire study population and in women, but not in men (eTable 6). When comparing patients with high-risk PFO with those without, we detected no difference in the association between PSS score as a discrete measure and early-onset CIS (in both groups, *p* value <0.001).

## Discussion

In this multicenter case-control study, we detected a robust independent association between self-perceived stress and early-onset CIS. This connection held true in young women, even after accounting for various well-known vascular factors, such as hypertension, physical inactivity, obesity, and current smoking. Of interest, we found no association between self-perceived stress and early-onset CIS in men.

This analysis adds to previous knowledge from earlier studies of associations between psychosocial stress and stroke by demonstrating that self-perceived stress is strongly correlated with IS,^27,28^ extending the evidence to early-onset CIS and using a validated stress measure, PSS. While PSS has been widely used to measure stress in various populations,^29,30^ our study is among the first to use it exclusively with CIS. Previously, self-perceived stress has been linked with increases of up to 4 to 8 times in the IS risk in young patients.^9,10^ However, most of these studies included young patients with IS with any type of etiology, in contrast to the specific subgroup of patients with CIS in our study.

Moreover, these studies also used different instruments to measure stress, making direct comparisons with our findings more challenging. In a large population-based prospective cohort study from the United Kingdom, 1-SD decrease in the Mental Health Inventory-5 (MHI-5) scale score was associated with an increased risk of stroke after adjustment for vascular risk factor with an OR of 1.11.^31^ The association remained significant for both women and men. The MHI-5 is a 5-item questionnaire representing psychological well-being in the past 4 weeks. It must be noted again that the study was inclusive of all stroke subtypes, whereas our study focuses specifically on the CIS. In another prospective community-based study, a 30-item General Health Questionnaire (GHQ) was used to measure psychological distress, defining presence of distress with a GHQ score of ≥5.^32^ This study had recruited only men aged 45–59 years and included IS of any type. Men with a GHQ score of 5 or greater had an increased risk of IS, with an OR of 1.41 in the fully adjusted model. Despite these methodological differences, the studies still identified significant associations between stress and stroke incidence, suggesting that stress, regardless of how it is measured, may play a key role in stroke pathophysiology.

One potential explanation for the higher self-perceived stress in women could be related to societal and psychological factors, where women often report experiencing more chronic stress due to juggling multiple roles, such as work, family, and caregiving. By contrast, men may show a stronger association with other risk factors, such as heavy alcohol consumption, which has been previously linked to an increased risk of stroke.^7^ Furthermore, men's lower stress scores may reflect societal conditioning to under-report stress, introducing potential bias in self-perceived stress measurements.

Psychosocial stress mechanisms linked to CIS are complex and not fully understood, but several pathways are plausible. Prolonged exposure to high levels of psychological stress is associated with chronic inflammation, endothelial dysfunction, platelet activation and aggregation, and autonomic dysregulation.^33^ Acute psychological stress may trigger qualitative changes in several procoagulant factors such as fibrinogen, factor XII, factor VII, factor VIII, von Willebrand factor, platelet activity, thrombin-antithrombin complexes, fibrin D-dimer, and tissue-type plasminogen activator.^34^ The resulting procoagulant activity outweighs the profibrinolytic response, promoting a hypercoagulable state. Similarly, chronic emotional stress and psychiatric conditions perpetuate hypercoagulation by enhancing procoagulant activity while diminishing fibrinolytic function.^34^ Other potential mechanisms might include acute repeated short-term spikes in blood pressure, vasospasm, and arrhythmias associated with stress. In addition, stress is often associated with behaviors such as smoking, physical inactivity, poor diet, and substance use, which might further elevate stroke risk. However, even after controlling for many of these lifestyle factors, the observed associations largely persisted in our analysis.

Earlier studies have also shown that many of the positive psychological traits, including optimism, a sense of purpose, environmental mastery, perceived rewards from social roles, and resilient coping mechanisms, have been linked to improved cardiovascular health. By contrast, higher levels of psychosocial stress and depression are strongly associated with adverse cardiovascular outcomes.^35^ This relationship is particularly relevant in the context of stroke because stroke is a significant complication of poor cardiovascular health.

One of the key strengths of the SECRETO study is the rigorous adherence to its prespecified and published study protocol, alongside the comprehensive and timely diagnostic assessments conducted for each participant. To ensure the homogeneity of the study population and eliminate stroke mimics, only patients with imaging-confirmed IS were included. In addition, all participants underwent standardized examinations, and data were collected through validated, structured questionnaires with high granularity. The number of missing data points was minimal because patients and controls were personally interviewed. Sensitivity analyses, such as limiting the control group to population-based individuals, further reinforced the robustness of the findings. The ability to adjust for multiple relevant potential confounders in the multivariable analyses also strengthens the study's validity. Furthermore, with participants recruited from 19 centers across Europe, the results are considered generalizable to populations of European origin.

Nonetheless, certain limitations should be considered. While the goal was to recruit all consecutive patients, some degree of selection bias may have occurred, for example, toward patients with milder strokes. However, previous research has shown that young patients with IS generally present with lower NIHSS scores compared with older individuals.^36^ Another important consideration is that the retrospective evaluation of prestroke stress conducted after the stroke may introduce recall bias. Some selection bias may have also affected the enrollment of controls because those experiencing higher levels of self-perceived stress might have been less interested to participate, possibly leading to an overestimation of the observed effect size. Our sensitivity analysis, however, indicated that even when limiting the sample to population-based controls, the effect size of PSS score remained similar and significant, despite a smaller sample size. One limitation of this study is the absence of data on early life stress or adverse childhood experiences, which are known to influence self-perceived stress levels in early adulthood. Evidence suggests that adverse childhood experiences can contribute to heightened stress perception through the cumulative impact of ongoing stressors, aligning with the stress proliferation hypothesis.^37^ Because the data for this study were primarily collected during hospitalization or shortly after discharge, we were also unable to determine whether the classification of stroke etiology in patients initially diagnosed with CIS later changed to a known cause. This limitation arises because of the lack of prolonged follow-up investigations, such as extended ECG monitoring, which could have identified undetected arrhythmias, including atrial fibrillation, as potential stroke mechanisms.

Our multicenter case-control study found higher self-perceived psychosocial stress to be associated with early-onset CIS, independent of traditional stroke risk factors. Subgroup analyses reinforced this link in women, although it was not observed in men. Further research is needed to delve deeper into the mechanisms that heighten the risk of early-onset CIS in individuals experiencing self-perceived stress, particularly focusing on the impact on the coagulation system and related pathways. Given the potential importance of CIS in the context of early-onset strokes, understanding the role of self-perceived stress in this population may be crucial for developing more effective prevention strategies.

## Glossary

CIS: cryptogenic ischemic stroke
GHQ: General Health Questionnaire
IQR: interquartile range
IS: ischemic stroke
MA: migraine with aura
NIHSS: NIH Stroke Scale
OR: odds ratio
PFO: patent foramen ovale
PSS: Perceived Stress Scale
TCD: transcranial Doppler
TEE: transesophageal echocardiogram
